# Cytotoxicity Study on Luminescent Nanocrystals Containing Phospholipid Micelles in Primary Cultures of Rat Astrocytes

**DOI:** 10.1371/journal.pone.0153451

**Published:** 2016-04-20

**Authors:** Tiziana Latronico, Nicoletta Depalo, Gianpiero Valente, Elisabetta Fanizza, Valentino Laquintana, Nunzio Denora, Anna Fasano, Marinella Striccoli, Matilde Colella, Angela Agostiano, M. Lucia Curri, Grazia Maria Liuzzi

**Affiliations:** 1 Dipartimento di Bioscienze, Biotecnologie e Biofarmaceutica, Università degli Studi di Bari Aldo Moro, Bari, Italy; 2 Consiglio Nazionale delle Ricerche, Istituto per i Processi Chimico-Fisici, Bari, Italy c/o Dipartimento di Chimica, Università di Bari, Bari, Italy; 3 Dipartimento di Chimica, Università degli Studi di Bari Aldo Moro, Bari, Italy; 4 Dipartimento di Farmacia – Scienze del Farmaco, Università degli Studi di Bari Aldo Moro, Bari, Italy; Brandeis University, UNITED STATES

## Abstract

Luminescent colloidal nanocrystals (NCs) are emerging as a new tool in neuroscience field, representing superior optical probes for cellular imaging and medical diagnosis of neurological disorders with respect to organic fluorophores. However, only a limited number of studies have, so far, explored NC applications in primary neurons, glia and related cells. Indeed astrocytes, as resident cells in the central nervous system (CNS), play an important pathogenic role in several neurodegenerative and neuroinflammatory diseases, therefore enhanced imaging tools for their thorough investigation are strongly amenable. Here, a comprehensive and systematic study on the *in vitro* toxicological effect of core-shell type luminescent CdSe@ZnS NCs incorporated in polyethylene glycol (PEG) terminated phospholipid micelles on primary cultures of rat astrocytes was carried out. Cytotoxicity response of empty micelles based on PEG modified phospholipids was compared to that of their NC containing counterpart, in order to investigate the effect on cell viability of both inorganic NCs and micelles protecting NC surface. Furthermore, since the surface charge and chemistry influence cell interaction and toxicity, effect of two different functional groups terminating PEG-modified phospholipid micelles, namely amine and carboxyl group, respectively, was evaluated against bare micelles, showing that carboxyl group was less toxic. The ability of PEG-lipid micelles to be internalized into the cells was qualitatively and quantitatively assessed by fluorescence microscopy and photoluminescence (PL) assay. The results of the experiments clearly demonstrate that, once incorporated into the micelles, a low, not toxic, concentration of NCs is sufficient to be distinctly detected within cells. The overall study provides essential indications to define the optimal experimental conditions to effectively and profitably use the proposed luminescent colloidal NCs as optical probe for future *in vivo* experiments.

## Introduction

Delivery of therapeutic agents in specific brain areas is a major challenge for the treatment of most neurological disorders. The blood-brain barrier (BBB) with its neuroprotective role hinders distribution of many relevant diagnostic and therapeutic agents in the brain parenchyma. Therapeutic molecules, antibodies and genes that may be potentially beneficial in the diagnosis and therapy of several neurodegenerative diseases, cannot, in fact, often cross the BBB in adequate amounts. Nanotechnology can have a significant clinical impact in neuroscience as it provides alternative tools for delivery of drugs and other molecules to specific targets in the Central Nervous System (CNS), and for new therapeutic applications [1–3]. In the last decade, many activities potentially able to revolutionize diagnosis and treatment of neurological diseases, have been developed in the field of nanomaterials [4,5]. In this perspective, neuroscience specific applications of emitting semiconductor colloidal nanocrystals (NCs) have been demonstrated relevant in cellular imaging and medical diagnosis of neurological disorders [6–8]. Luminescent NCs display unique and superior optical properties compared with traditional organic fluorescent dyes, such as broadband excitation, narrow bandwidth emission, high quantum yield, resistance to quenching and high photochemical stability [9]. In addition, due to their small size, designed fluorescent NCs could effectively interact with neuronal and glial cells of CNS, at cellular and subcellular levels, resulting good candidates for tracking studies of molecular dynamics of intra or intercellular process. Also, more complex and advanced NC-based systems may further provide powerful nanoplatforms for investigating the effects of drugs or other molecules employed for the care and treatment of neurological diseases [10,11]. Despite the growing literature on the use of luminescent NCs as imaging and diagnosis agents on a wide variety of cell types, typically tumor cells or immortalized cells, only a limited number of studies have so far explored their applications in primary neurons, glia and related cells [10,12]. Therefore, approaches for an early and sensitive detection of glia and neuron response to luminescent NCs are needed. Astrocytes are the major glial cell type in the brain and their activation is one of the key components of the cellular responses to stress and brain injuries. Therefore, astrocytes may represent a useful target to study the interaction between NCs and CNS cells. Indeed, understanding and evaluation of the possible cytotoxicity of colloidal NCs on *in vitro* systems is a pre-requisite for their *in vivo* use in the clinical field [13]. Indeed, the widespread biological use of luminescent NCs is severely limited by the presence of a core containing cadmium as inorganic component, suspected of causing biological toxicity at both cellular and tissue level, as a result of their degradation in the biological environment. There are, in fact, well-documented cytotoxic effects of cadmium and other heavy metal ions [14–17]. Using the MTT assay, Derfus et al. [18] revealed for the first time, in an *in vitro* system, that CdSe NCs can be degraded in response to UV irradiation, thus causing cell death due to the release of cadmium. Therefore, while a proper functionalization of NC surface is essential in order to limit the toxicity, preliminary studies assessing the toxicity of the resulting nanostructure *in vitro* in cell culture systems before their use for clinical purposes in any animal subject is fundamental.

A successful strategy of NC surface protection is based on their incorporation in the hydrophobic core of polymer grafted lipid micelles. Generally, polymeric micelles represent nano-formulations that ensure good biocompatibility and flexibility in terms of design modification. Interestingly, the approach based on encapsulation of colloidal NCs in lipid micelles allows the further co-incorporation, in the hydrophobic core, or the conjugation, on the surface, of a wide range of drugs. In addition, the micelle surface can be also conjugated with targeting ligands able to recognize specific molecular markers, allowing receptor-mediated endocytosis and, thus, improving therapeutic efficacy of drugs. Therefore, starting from suitably functionalized NCs, enhanced drug delivery nanoplatforms could be designed and obtained, thus combining the targeted therapeutic activity of the drugs with the original size dependent optical properties of the luminescent NCs, ultimately acting as diagnosis probes, potentially able to cross the BBB with their payload.

On the basis of these considerations, this work aimed at evaluate *in vitro*, in primary cultures of rat astrocytes, the toxicity of polyethylene glycol (PEG)-terminated phospholipid micelles embedding luminescent NCs, namely core shell nanostructures consisting of a cadmium sulphide core and a zinc sulphide shell (CdSe@ZnS). The PEG termination providing a hydrophilic protective layer at the NC containing micelle surface, was demonstrated effective for limiting the natural blood opsonization process of the particles, further preventing the recognition by macrophages and thus increasing their half-life in blood. In addition the ability of PEG-terminated nanoparticles to penetrate the CNS of animals with early symptoms of the neuroinflammatory diseases was been reported [19].

Overall, the obtained luminescent NC containing PEG-terminated micelles are good candidates for the future *in vivo* studies.

In this work, the cytotoxicity of cadmium based luminescent NCs was investigated before and after their incorporation in PEG modified lipid micelles. In addition, cytotoxic behavior of the empty PEG-modified micelles was compared to the NC containing ones, in order to discriminate the toxicity contribution ascribed to the inorganic NCs from that due just to the organic molecules capping the NC surface, and thus obtain relevant insight on possible different responses affecting cell viability. Furthermore, the effect of charge and end group composition at nanoparticle surface, which are also known to influence the cell interaction and response, on the cytotoxicity of astrocytes, was investigated by using different PEG-modified phospholipid micelles, namely without terminal groups and with two distinct functional end groups, amine and carboxyl groups, respectively.

Finally, the ability of PEG-lipid micelles to penetrate the cells was qualitatively assessed by fluorescence and confocal microscopy investigation and quantitatively demonstrated by photoluminescence (PL) assay. The overall results clearly indicated that, even at low concentration, NCs incorporated into the micelles can be satisfactorily detected within the cells. A suitably designed set of experiments was performed to define the optimal concentrations and appropriate conditions for their *in vivo* use.

## Material and Methods

### Ethics statement

All experimental procedures involving animals were carried out in strict accordance with the recommendations in the NIH Guide for the Care and Use of Laboratory Animals and approved by the Institutional Animal Care and Use Committee of University of Bari, Italy (Permit Number: 23-98-A). All efforts were made to minimize the number of animals used and to ameliorate their suffering.

### Chemicals

All chemicals were purchased with the highest purity available and used as received without any further purification or distillation. Cadmium oxide (CdO, powder 99.5%), selenium (Se, powder 99.99%), oleic acid (OLEA, technical grade 90%), trioctylphosphine oxide (TOPO, 99% and technical grade), tributylphosphine (TBP, 99%), trioctylphosphine (TOP, technical grade), t-butylphosphonic acid (98%), diethylzinc (Et_2_Zn), hexamethyldisilathiane (HMTS) and phosphotungstic acid (99.995%) were purchased from Aldrich. Hexadecylamine (HDA) was purchased from Fluka.

1,2-Dipalmitoyl-sn-glycero-3-phosphoethanolamine-N-[methoxy(polyethylene glycol)-2000] (PEG-2-PE), 1,2-distearoyl-snglycero- 3-phosphoethanolamine-N-[carboxy(polyethylene glycol)-2000] (DSPE-PEG-COOH) and amine 1,2-distearoyl-sn-glycero-3-phosphoethanolamine-N-[amino(polyethylene glycol)-2000] (DSPE-PEG-NH_2_) were purchased from Avanti Polar Lipids.

Dulbecco’s modified Eagle’s medium (DMEM), fetal bovine serum (FBS), penicillin and streptomycin were obtained from GIBCO (Paisley, Scotland). DNase 1, poly-L-lysine (PLL), trypsin, Trypan Blue, and 3-(4,5-dimethylthiazol-2-yl)-2.5-diphenyltetrazolium bromide (MTT) were provided by Sigma (St. Louis, MO, USA). 2′,7′-dichlorofluorescein diacetate (DCFH-DA) was obtained from Calbiochem. Anti glial fibrillary acidic protein (GFAP) antibodies were purchased from Serotec (Oxford, UK).

All solvents used were of analytical grade and purchased from Aldrich. All aqueous solutions were prepared by using water obtained from a Milli-Q gradient A-10 system (Millipore, 18.2 MΩ cm, organic carbon content ≤4 μg/L).

### Synthesis of CdSe@ZnS nanocrystals

Core-shell structures of CdSe@ZnS NCs were synthesized following a literature reported approach with minor modification [20]. In particular, the core synthesis was achieved by introducing CdO (1.127 g), as cadmium precursor, and a mixture of hexadecylamine (12.00 g, HDA), trioctyl-phosphine oxide (12.00 g, TOPO) and t-butylphosphonic acid (0.28 g) in a three-neck flask, warming up under inert atmosphere until TOPO and HDA melt, and then degassing for 1h. The temperature was then risen up to 300°C and a mixture of selenium (0.39 g, Se) and tributyl-phosphine (4.40 g) was injected to start the formation of CdSe nuclei; their subsequent growth was carried out at 270°C. The CdSe NC growth was stopped by cooling down the reaction mixture to 100°C and let it stir for 1 hour for thermal annealing. The shell growth was carried out by a drop-wise injection of a stock solution of ZnS precursors containing TBP, Et_2_Zn (1M solution in heptane) and HMTS in the same reaction flask at 155°C. CdSe@ZnS NCs, as red powder, were then collected by centrifugation after the addition of methanol as a non-solvent and further dispersed in CHCl_3_.

### Preparation of PEG-lipid micelles with different surface end groups

Empty PEG-lipid micelles bearing different surface end groups (MIC, MIC-COOH and MIC-NH_2_) were prepared by dissolving PEG-modified phospholipids in CHCl_3_. Namely, empty PEG-lipid micelles without functional groups (MIC) were obtained by using only PEG-2-PE (100%), at a defined concentration (2.25*10^−3^ M), far above its critical micelle concentration (CMC) value (~ 10^−6^ M). Conversely, empty PEG-lipid micelles with terminal amine groups (MIC-NH_2_) were prepared by using a CHCl_3_ mixture containing PEG-2-PE (80% w/w) and DSPE-PEG-NH_2_ (20% w/w). Likewise, empty PEG-lipid micelles with terminal carboxyl groups (MIC-COOH) were achieved starting from PEG-2-PE (80% w/w) and DSPE-PEG-COOH (20% w/w) dispersed in CHCl_3_. The resulting final concentration of PEG-modified phospholipids was fixed at 2.25*10^−3^ M in each sample. Anyhow, a dried PEG-lipid layer was attained by CHCl_3_ evaporation under nitrogen flux and then kept under vacuum for 1 h. Subsequently, the formation of PEG-lipid micelles was promoted by addition of aqueous PBS buffer (10 mM at pH 7.4) to the dried lipid films and by heating samples to 80°C. The samples were, indeed, equilibrated at the relevant temperatures for 20 min, with intermittent vigorous vortex mixing, and were thereafter allowed to cool to room temperature during 20 min before the cycle was repeated (3 cycles) [21,22]. Finally, PEG-lipid micelles were purified by centrifugation (13000 x g for 5 min) and filtration (0.2 μm filters, Anotop, Whatman).

### Encapsulation of CdSe@ZnS nanocrystals in PEG-lipid micelles

PEG-modified phospholipid micelles with terminal carboxyl groups (NC/MIC-COOH) were obtained by following the same protocol used for the preparation of empty micelles, just introducing defined amounts of CdSe@ZnS NCs in the CHCl_3_ mixture containing 80% PEG-2-PE and 20% DSPE-PEG-COOH. The NC concentration in the PEG-lipid micelles was varied starting from different amount of stock solution of *‘as synthesized’* NCs in CHCl_3_ [21,22]. The NC concentration was evaluated from the absorbance spectra, by using the extinction coefficient as previously reported [23].

### Preparation of astrocyte cultures

Astrocytes were prepared from primary cell cultures of neocortical tissues from 1-day-old rats as described by Latronico et al. [24]. For our experiments we used 6 litters of 12 pups each. The pups used were from six female Wistar rats which were breeding and mated in the animal facility of the Department of Biosciences, Biotechnologies and Biopharmaceutics of University of Bari (Italy). All animals were maintained on a 12 h: 12 h light/dark cycle; room temperature was kept at 22°C and humidity was controlled at 40–50%. Each pregnant dam was housed individually in clear plastic cages with free access to food and tap water. One day after birth the pups were removed from their dams, anesthetized with ether vapors, then sacrificed by rapid decapitation and the dissected neocortical tissues were used for the preparation of primary glial cell cultures as described Latronico et al. [24]. Then, brains were cleaned of meninges and blood vessels, and the dissected neocortical tissues were minced by passage through a stainless steel mesh (40 mesh) and incubated with 0.25% trypsin and 0.01% DNase in DMEM for 10 min at 37°C. After addition of FBS, the dissociated cells were passed through a 100 mesh and viability of cells was assessed by Trypan Blue dye exclusion. Cells were plated in PLL-coated flasks (75 cm^2^) at a density of 1.5x10^7^ viable cells/flask in DMEM, 100 U/ml penicillin, 100 μg/ml streptomycin, 10% FBS and maintained at 37°C in a 5% carbon dioxide incubator with a renewal of medium twice a week. After 7–10 days in culture, microglia and oligodendrocytes were separated from astrocytes by mechanical dislodging and then the astrocytes were obtained by trypsinization (0.25% trypsin/0.02% EDTA) [25]. Astrocytes were purified by three cycles of replating and trypsinization in order to deplete cultures of microglia and oligodendrocytes. The purity of the final cell culture was assessed by immuno-staining for GFAP. More than 98% of the cells were GFAP-positive in all the preparations.

### MTT cell viability assay

Cytotoxicity on astrocyte cells of empty micelles and luminescent NCs, before and after functionalization with PEG-modified phospholipid micelles, was detected using MTT [3-(4,5-dimethylthiazol-2-yl)-2,5-diphenyl tetrazolium bromide] assay [26].

This assay is based on the reduction of MTT by the mitochondrial succinate dehydrogenase in viable cells, to a blue formazan product which can be spectrophotometrically measured by using a microplate reader.

This assay was specifically identified in order to characterize the NP concentration range toxic for the cells, as, differently from other colorimetric or fluorescent dyes used to detect just cell viability in cultured cells, it allows to distinguish between healthy cells and cells that, though still alive, already lost their vital functions.

Briefly, after treatment for 24 h with *‘as synthesized’* NCs or empty micelles (MIC, MIC-COOH MIC-NH_2_) or NC/PEG-lipid micelles (NC/MIC, NC/MIC-COOH and NC/MIC-NH_2_), the culture medium was removed and cells were rinsed with PBS and incubated at 37°C, 5% CO_2_ for 2 h with 0.5 mg/mL MTT. Reaction was stopped by removing the medium and the formazan crystals in the cells were solubilized with absolute ethanol. The absorbance values at 560 and 690 nm were recorded by means of a VersaMax Microplate Reader (Molecular Devices, Sunnyvale, CA, USA). The difference between the absorbance of each sample at 560 and 690 nm was measured. The value of the untreated sample (control, CTRL) was set at 100% and the cell viability was expressed as percentage of CTRL. The value of cytotoxicity was also expressed as IC50 that is the concentration necessary for 50% inhibition in the same MTT essay.

### Reactive oxygen species detection

Detection of intracellular free radicals was performed by loading astrocytes (in 6-well plates) with 10 μM 2′,7′-dichlorofluorescein diacetate (DCFH-DA) in phenol red—free DMEM at 37°C for 30 min [27]. Then wells were washed twice with DMEM and treated with NCs, MIC-COOH or NC/MIC-COOH. In particular, cells were treated for 1 h and 30 min with: NC at the final concentrations of 1.8 and 35 nM, NC/MIC-COOH at the final phospholipid concentrations of 5 and 100 μM (corresponding at NC concentration of 1.8 and 35 nM) and MIC-COOH at the same phospholipid concentrations (5 and 100 μM). Cells treated only with DCFH-DA or with 100 μM of H_2_O_2_ represented the negative (CTRL) and positive controls, respectively. After incubation, the culture medium was removed and cells were rinsed three times with PBS to remove excess of NCs, NC/MIC-COOH or MIC-COOH. Cells were lysed with Tris-HCl 10 mM/NaCl 150mM/Triton X-100 0.5%, pH 7.5, then centrifuged at 10.000 x g, 4°C for 10 min. Supernatants were collected and their spectrofluorometric analysis was performed at 525 nm under excitation at 485 nm. Results were normalized to total proteins and Reactive Oxygen Species (ROS) production was expressed as a relative percentage of photoluminescence (PL) intensity with respect to a negative control.

### Intracellular localization of NC/MIC-COOH nanoparticles by fluorescence microscopy

*Epifluorescence Microscopy* Live cell fluorescence measurements were performed on coverslips with NC/MIC-COOH-loaded astrocytes mounted in an open-topped perfusion chamber (Series 20, Warner Instrument Corp, Hamden, CT) using an inverted microscope (Nikon Eclipse TE2000-U microscope) equipped for single cell fluorescence measurements [28]. To optimize emission and reduce the exposure time in the imaging setup, samples were excited at 410 nm with a monochromator (DeltaRam V, PTI) through a 40X oil immersion objective (NA = 1.30) and the emitted fluorescence, filtered through a BFP long pass filter (435 ALP, Omega Optical, Brattleboro, VT, USA) was detected by a cooled CCD camera (CoolSNAP HQ, Photometrics). Fluorescence measurements were carried out using the Metafluor software (Molecular Devices, MDS Analytical Technologies, Toronto Canada). Brightness, contrast adjustments and necessary cropping were performed using the NIH IMAGEJ program. Images were acquired in living astrocytes cultured on polylisine-coated coverslips and incubated for 9 h in serum free DMEM containing NC/MIC-COOH at the final phospholipid concentration of 200 nM. After incubation, the culture medium was removed and cells were perfused with an extracellular Ringer's solution [29].

*Confocal Microscopy* Laser scanning confocal microscopy was performed on a Leica TCS SP8 X (Leica Microsystems, Germany) inverted confocal microscope using a ×63, 1.40 numerical aperture oil immersion lens for imaging. Laser beams with 405 nm and 488 nm excitation wavelengths were used for Hoechst and NC imaging, respectively. Differential interference contrast (DIC) microscopy has been used to highlights cellular boundary.

Confocal images were acquired by using sequential scanning to exclude possible cross-talks. Moreover, images were attained by using six line average to increase signal to noise response. All data were processed with Leica LAS AF LITE (Leica microsystems, Germany). Images were acquired on fixed cells. Namely astrocytes were exposed to NC/MIC-COOH at the final phospholipid concentration of 2 μM and NC concentration of 0.2 nM or to *‘as synthesized’* NCs at NC concentration of 0.2 nM. Following the incubation period of 1h, cells were washed two times with PBS, fixed with 4% formaldehyde in PBS (15 min) and finally treated with 2 μg/ml of Hoechst 33258 to stain cell nuclei (10 min). Cells were then rinsed three times with PBS and mounted with glycerol/PBS on glass slides [30].

### Quantitative analysis of nanoparticle uptake by spectrophotometric assay in cell lysates

Nanoparticle cellular uptake was measured by treating the astrocytes, cultured in 6-well plates, with NC/MIC, NC/MIC-NH_2_ or NC/MIC-COOH samples having a phospholipid and NC concentration of 2.7 mM and 2.9 μM, respectively. In particular, cells were treated for 1 h with MIC, NC/MIC-NH_2_ or NC/MIC-COOH at the final phospholipid concentrations of 5, 12.5, 25, 50, 100 and 200 μM, respectively. The tested NC concentrations were varied in the range from 0 to 214.8 nM. Untreated astrocytes were used as negative controls (CTRL). After incubation, the culture medium was removed and cells were rinsed three times with PBS to remove excess of nanoparticles. Cells were lysed with Tris-HCl 10mM/NaCl, 150mM/Triton X-100 5%, pH 7, then centrifuged at 13.000 x g. The concentration of fluorescent CdSe@ZnS NCs in cell lysates was determined by creating of a calibration curve. In particular, measurements of the photoluminescence (PL) intensity were recorded as a function of NC concentration by using solutions of NC/MIC, NC/MIC-NH_2_ or NC/MIC-COOH with known concentrations of phospholipids and NCs ranging from 0 to 200 μM and from 0 to 214.8 nM, respectively. Finally, the calibration curve was obtained by plotting the area under the curve of PL emission band in the wavelength range between 550 and 700 nm versus NC concentration.

### Photophysical characterization

Absorption and photoluminescence measurements were performed by means of a UV/Vis/NIR Cary 5 spectrophotometer (Varian) and the Eclypse spectrofluorimeter (Varian), respectively. The optical measurements on the CdSe@ZnS NC solution were carried out at room temperature on the organic solution obtained directly from synthesis without any size-sorting treatments. NC/micelles were investigated in PBS buffer at room temperature.

### Investigation on particle size, size distribution and colloidal stability

Size, size distribution and colloidal stability of the micelles were investigated by using a Zetasizer Nano ZS, Malvern Instruments Ltd., Worcestershire, UK (DTS 5.00). In particular, size and size distribution were determined by means of dynamic light scattering (DLS) measurement. Size distribution was described in terms of polydispersity index (PDI). The ζ-potential measurements were carried out by using a laser Doppler velocimetry (LDV) after sample dilution in KCl aqueous solution (1 mM). All reported data are presented as mean values ± standard deviation obtained from three replicates.

### Transmission electron microscopy characterization

Transmission Electron Microscopy (TEM) analysis was performed by using a Jeol Jem-1011 microscope, working at an accelerating voltage of 100 kV. TEM images were acquired by a Gatan Orius SC1000CCD Camera. The samples were prepared by dropping on the 400 mesh amorphous carbon-coated Cu grid a CdSe@ZnS NC CHCl_3_ dispersion and letting the solvent to evaporate. For the positive staining TEM observation, after the sample deposition, the grid was dipped in a 2% (w/v) phosphotungstic acid solution for 30 seconds. Staining agent excess was removed from the grid by rinsing with ultrapure water (dipping the grid in ultrapure water three times for 10 seconds). The sample on the grid was left to dry overnight and finally stored in a vacuum chamber until analysis.

### Statistical analysis

The statistical analyses were performed using GraphPad Prism 4 (GraphPad Software, San Diego California USA).

## Results and Discussion

**Fig 1:**
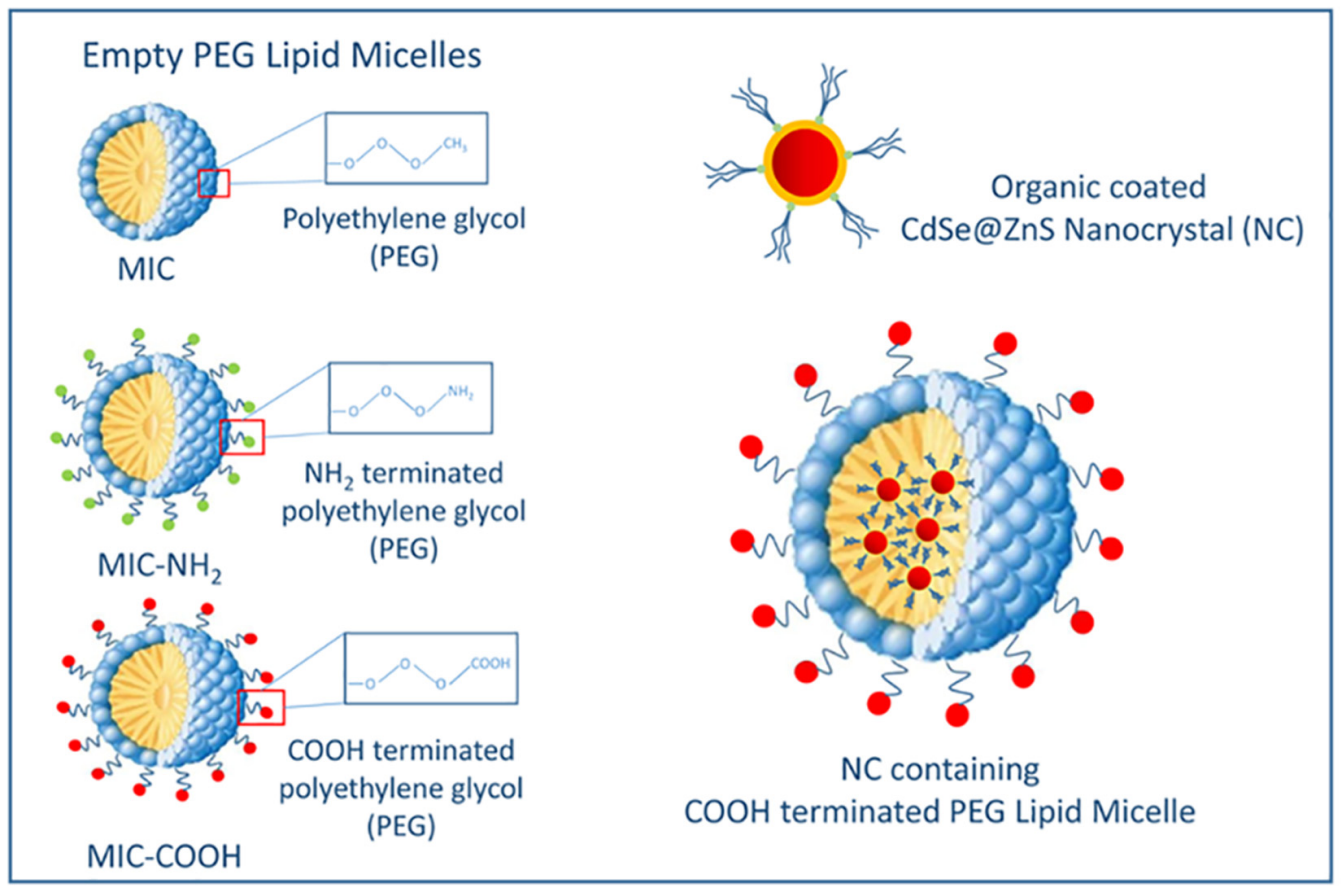
Schematic description of empty and NC containing PEG-modified lipid micelles without terminal groups and with COOH or NH_2_ moieties, respectively and corresponding short hand notation.

The toxicological effects of surface functionalized luminescent NCs were assessed in an *in vitro* system represented by primary cultures of rat astrocytes, which are the principal glial cell type in the brain and are known to play an important pathogenetic role in several neurodegenerative and neuroinflammatory diseases. In particular, the cytotoxicity on astrocytes of CdSe@ZnS NCs was investigated, before and after their incorporation in PEG-modified phospholipid micelles, by means of MTT cell viability assay and ROS detection. In addition, toxicity studies of empty PEG-lipid micelles were carried out, in order to specifically evaluate the effect of the PEG modified lipid molecules used to coat NC surface. As a matter of fact, the potential toxicity of nanomaterials mostly depends on their actual formulation. Therefore, an investigation of possible toxicity due just to the shell embedding NCs is essential, since composition of such a coating may affect the nanoparticle internalization across the cell membrane and their subsequent intracellular distribution. In addition, in order to investigate the effect of end groups and charge at the micelle surface, cytotoxicity studies were performed on empty PEG-lipid micelles formed of bare PEG-2-PE and of a mixture of PEG-2-PE and DSPE-PEG-NH_2_ or DSPE-PEG-COOH, respectively. Indeed, terminating group and charge at nanoparticle surface were reported to play a significant role on their interaction with cells and accordingly on cytotoxicity [31]. In particular, carboxyl (COOH) and amine (NH_2_) end groups, respectively, were selected as they cannot only impart a different charge to the micelle, but also be readily enable their further conjugation with biomolecules or drugs, thus resulting relevant for design and realization of targeting nanosystems. The list of the different samples tested in the cytotoxicity studies, namely empty and NC containing PEG-modified lipid micelles, further classified accounting for the chemical end group, is reported in Fig 1.

Finally, the cellular uptake of NC containing PEG-modified lipid micelles in astrocytes was both qualitatively and quantitatively assessed by means of two distinct methods, namely fluorescence microscopy analysis and emission spectroscopy determination in cell lysates.

### Assessment of organic capped CdSe@ZnS nanocrystal toxicity on cell viability of astrocytes

A first set of experiments was devoted to investigation of toxicity of *‘as synthesized’* luminescent organic capped CdSe@ZnS NCs, before their incorporation in phospholipid micelles.

**Fig 2:**
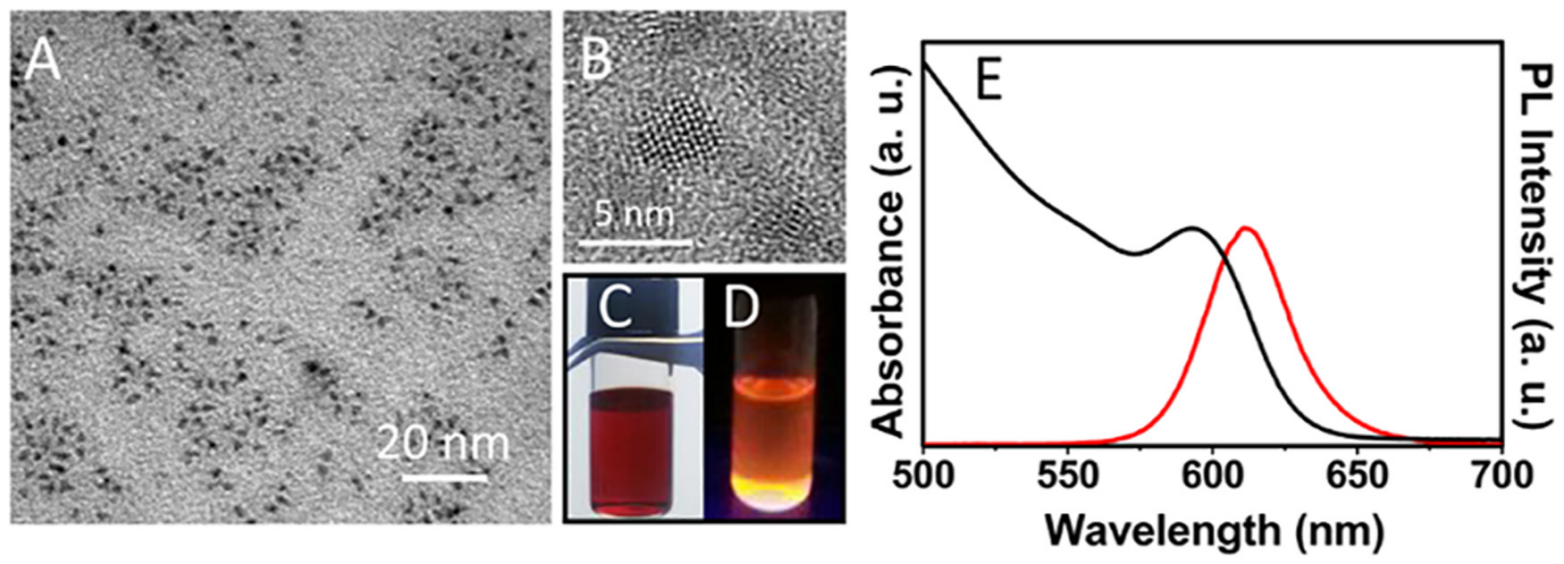
Preparation and characterization of *‘as synthesized’* CdSe@ZnS NCs. TEM (A) and HRTEM micrographs (B), absorption (E, black line) and PL (E, red line) spectra of organic capped CdSe@ZnS NCs dispersed in CHCl_3_. Picture of the sample under visible (C) and UV (D) light illumination.

Firstly, luminescent core-shell NCs were prepared starting from the synthesis of CdSe cores by decomposition of organometallic precursors in hot coordinating solvents, followed by *in situ* growth of ZnS shell, resulting in hydrophobic organic coated CdSe@ZnS NCs well dispersible in organic solvent (Fig 2C and 2D). In general, the growth on CdSe NCs of a higher band gap materials, such as an epitaxial shell of ZnS, is able not only to increase the chemical stability of the structure, but also confine the charge carriers inside the core, thus increasing the emission property of the resulting core-shell NCs.

The *‘as synthesized’* NCs were characterized by optical and morphological techniques. The absorption spectrum of luminescent CdSe@ZnS NC in CHCl_3_ shows a first excitonic peak centered at 593 nm, (Fig 2E black line), which corresponds to 4 nm sized particles as also confirmed by the TEM and HRTEM micrographs (Fig 2A and 2B) [23].

**Fig 3:**
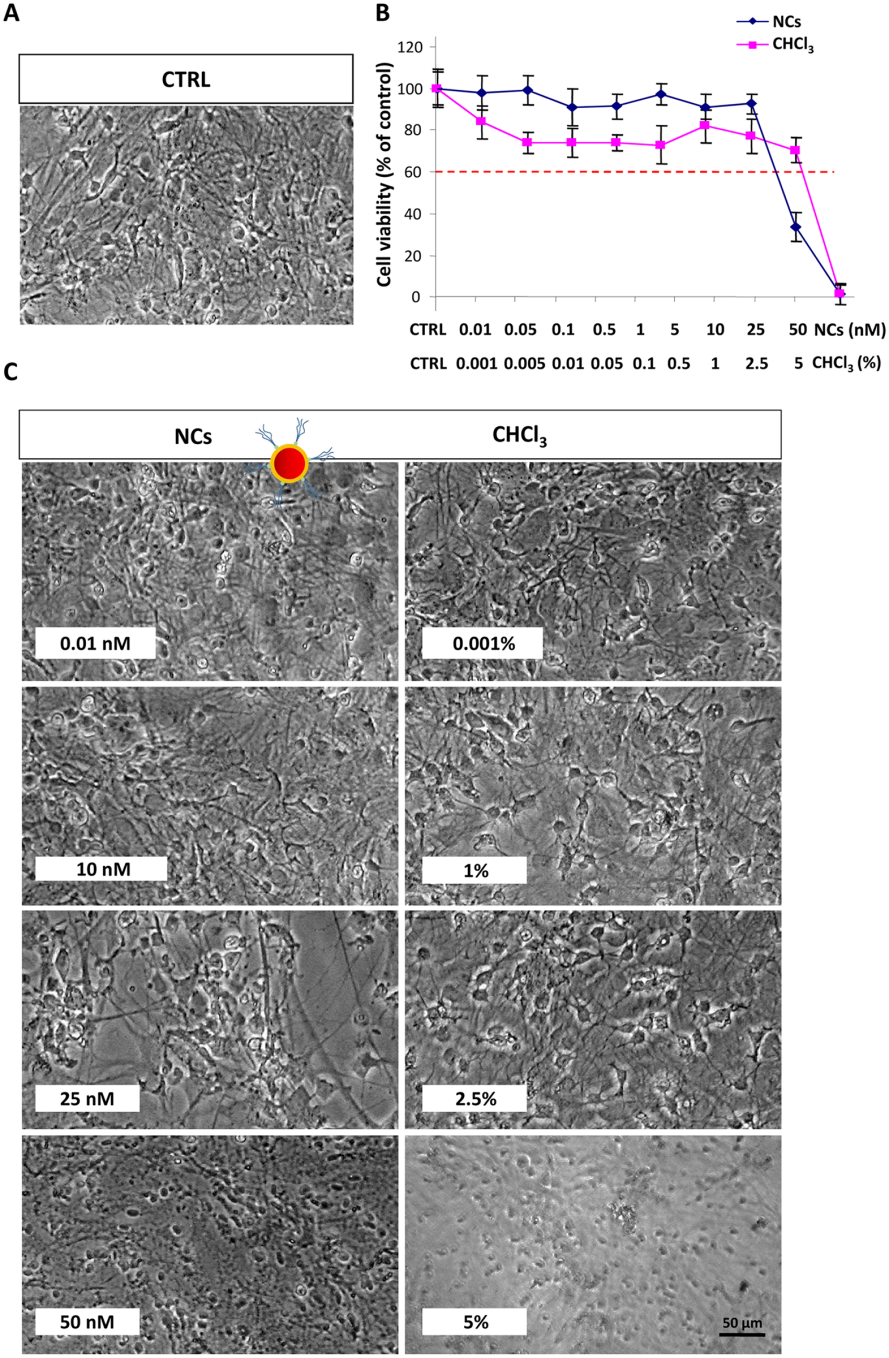
Effect of organic capped CdSe@ZnS NCs on cell viability of astrocytes. Confluent astrocytes, plated in 96 well plates, were treated with luminescent organic capped CdSe@ZnS NCs or CHCl_3_ at the indicated concentrations. The control (CTRL, A) was obtained from untreated astrocytes in serum-free DMEM. After treatment for 24 h at 37°C, 5% CO_2_ the cells were subjected to the cell viability test with MTT as described in Experimental section. Micrographs show representative results of cell morphology observed under phase-contrast microscope (50X magnification) after 24 h of treatment with NCs (C, left panel) or CHCl_3_ (C, right panel). The graphs represents the cell viability expressed as percentage of cell survival in comparison with control (CTRL) (B). A dose of NCs or CHCl_3_ that determined a cell viability < 60% was considered toxic. Data represent the mean values ± SD of three different experiments performed on different cell populations.

The PL spectrum of the same sample dispersed in CHCl_3_ clearly highlights an emission signal centered at 610 nm (Fig 2E, red line), and with a narrow bandwidth, which is characteristic of the highly size monodisperse sample. Subsequently, cytotoxicity of the *‘as synthesized’* luminescent NCs, was tested on astrocyte cells. For this purpose, confluent astrocytes were treated with different solutions of organic capped NCs diluted in culture medium at the final concentrations of 0.01, 0.05, 0.1, 0.5, 1, 5, 10, 25 and 50 nM. In addition, since NCs in the stock solution were dispersed in CHCl_3_, a dose-response curve to CHCl_3_, diluted in culture medium at the same solvent concentration present in the NC samples (namely CHCl_3_ content of 0.001% in the 0.01 nM NC solution, 0.005% in the 0.05 nM, 0.01% in the 0.1 nM, 0.05% in the 0.5 nM, 0.1% in the 1.0 nM, 0.5% in the 5.0 nM, 1% in the 10.0 nM, 2.5% in the 25.0 nM and 5% in the 50 nM), was run in each experiment to obtain a reliable estimation of NC toxicity. The negative control (CTRL, Fig 3A) was represented by untreated cells. As shown in Fig 3B, NCs were not found cytotoxic for astrocytes up to the concentration of 10 nM (IC50 = 25.54 nM, 95% confidence interval = 14.64–36.43 nM). CHCl_3_ did not exert cell toxicity at the corresponding concentration of 1%. Moreover, a cell viability higher than 60% was observed even upon exposure to 2.5% CHCl_3_ (IC50 = 3.75%, 95% confidence interval = 3%-5%), corresponding to the NC toxic dose of 25 nM. This evidence clearly indicates that the cell mortality, observed under such conditions, is dependent on the actual NC concentration, rather than on that of the CHCl_3_ used as dispersing medium. As shown in Fig 3 (panel C), astrocytes treated with NCs at a concentration up to 10 nM as well as those treated with same amount of bare CHCl_3_, showed a morphology similar to that of control cells.

Conversely, cells treated with 25 nM NC were observed reducing in number, with concomitant signs of cell suffering, whereas astrocytes treated with the corresponding dose of bare CHCl_3_ (2.5%) showed a morphology comparable to that of control cells. At a 50 nM NC and at the corresponding concentration of bare CHCl_3_ (5%), astrocytes were found devoid of branches, with a not defined cell body.

The observed NC cytotoxicity at the highest tested concentrations can be, in principle, ascribed to different factors. Although *‘as synthesized’* CdSe@ZnS NCs were reported able to enter cells by endocytosis, by means of passive and non-specific mechanisms, finally inducing mitochondrial dependent apoptotic processes [32,33], here, from experiments performed on cell lysates, NCs were found not to enter the cells. Therefore their toxicity can be assumed as ascribed to release of Cd^+2^ ions. It has been widely reported that, irrespectively from the ability of CdSe@ZnS NCs to enter cells, when their surface is not suitably functionalized, they are unstable and metal ions can be released from the nanoparticles [34] thus resulting toxic due to an intracellular ROS production induced by heavy metal ions [35,36]. Furthermore, the bare NCs have been demonstrated not only able to release of Cd^+2^ ions, but also to precipitate at cell surface, thus impairing cell function and leading to the death [32]. The ensemble of considerations clearly points out that the ZnS shell does not completely prevent the release of the Cd^+2^ ions, and highlights the need to protect NC surface from deterioration in biological media in order to mitigate their toxicity. Therefore, here incorporation of CdSe@ZnS NCs in the hydrophobic core of PEG-modified phospholipid micelles was performed.

### Effect of empty PEG-lipid micelles on cell viability of astrocytes

**Fig 4:**
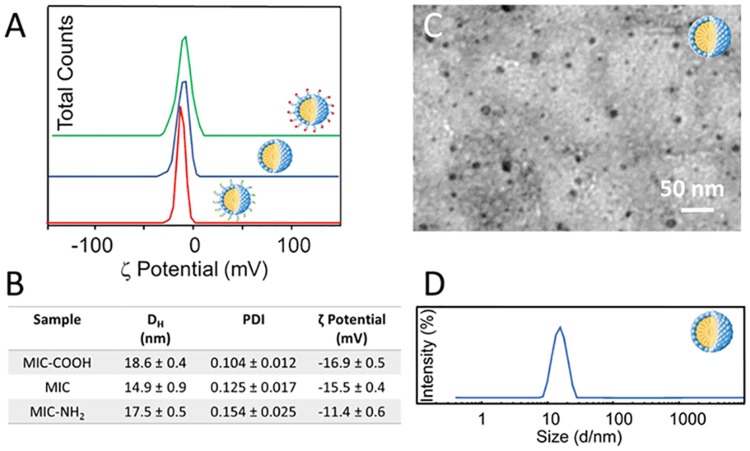
**ζ-Potential measurements of empty PEG-modified lipid micelles without terminal groups (MIC, blue line) and with COOH (MIC-COOH, green line) or NH**_**2**_ **groups (MIC-NH**_**2**_**, red line) (A).** Size distribution of empty PEG-modified lipid micelles described in terms of average hydrodynamic diameter by intensity and polydispersity index (PDI). Surface charge of empty PEG-modified lipid micelles by ζ-potential measurements (B). TEM micrograph with positive staining of MIC (C). Hydrodynamic diameter distribution by intensity of MIC dispersed in PBS at pH 7.4 (D).

While the need of a suitable NC surface functionalization is now obviously motivated, on the other hand, an assessment of possible cytotoxicity of the surface layer protecting and functionalizing NCs remains essential. In fact, toxicity could be induced not only by the inorganic NC itself but also by the NC surface-coating molecules [32]. Therefore, since CdSe@ZnS NCs were incorporated in the hydrophobic core of PEG-modified phospholipid micelles, the toxicity of empty PEG-lipid micelles was preliminary examined. In addition, cytotoxicity was investigated as a function of the chemistry of the different functional end groups of the micelles and, accordingly, of their overall charge. Therefore, bare empty PEG-modified lipid micelles, without any functional end group (MIC), with COOH (MIC-COOH) and NH_2_ end groups (MIC-NH_2_), respectively, were prepared (Scheme 1) and characterized by DLS analysis, TEM investigation and ζ-potential measurements (Fig 4).

**Fig 5:**
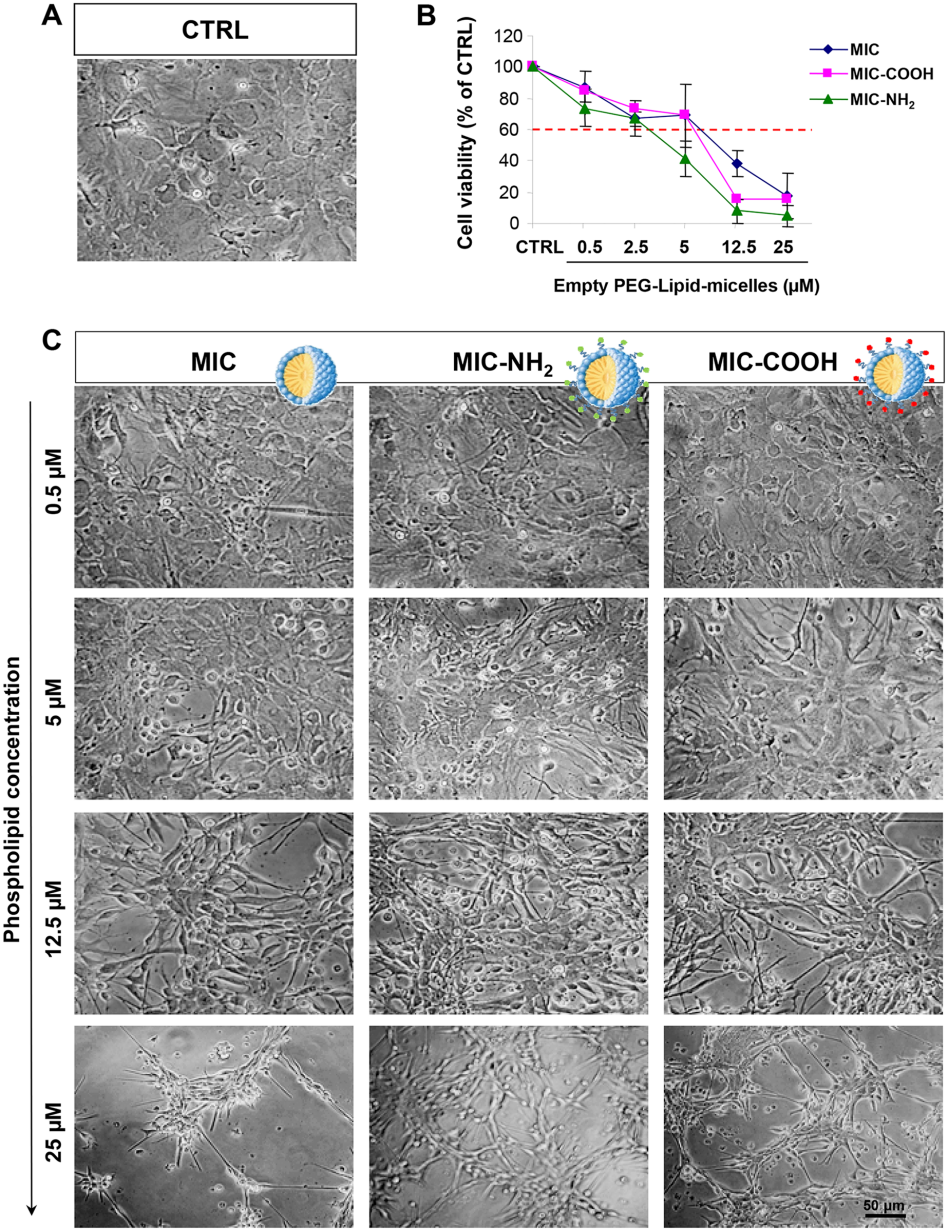
Effect of empty PEG-lipid micelles on cell viability of astrocytes. Confluent astrocytes, plated in 96 well plates, were treated with the three different preparation of PEG-lipid micelle represented by PEG-lipid micelle, bare, i. e. without terminal groups (MIC), and COOH- (MIC-COOH) or NH_2_- (MIC-NH_2_) terminated, respectively, at the indicated phospholipid concentration. The control (CTRL) was obtained from untreated astrocytes in serum-free DMEM. After treatment for 24 h at 37°C, 5% CO_2_ the cells were subjected to the cell viability test with MTT as described in Experimental section. Micrographs show representative results of cell morphology observed under phase-contrast microscope (50X magnification) after 24 h of treatment. The graphs represent the cell viability expressed as percentage of survival cells in comparison with control (CTRL). The dose of MIC, MIC-COOH and MIC-NH_2_ determining a cell viability < 60% was considered toxic. Data report the mean values ± SD of three experiments performed on different cell populations.

DLS investigation performed on empty PEG-modified lipid micelles reveals a monomodal size distribution (Fig 4B and 4D). In Fig 4D, the size distribution of MIC is reported, indicating an average hydrodynamic diameter of 14.9 ± 0.9 nm. Similar trends in size distribution can be observed for the MIC-COOH and MIC-NH_2_ samples (data not shown) resulting in average hydrodynamic diameter reported in Table B in Fig 4. Such an observation is further supported by TEM investigation performed on MIC cast on TEM grid after positive staining with phosphotungstic acid, revealing the formation of micelles having an average size of about 15 nm (Fig 2B). Also in this case, similar results were obtained for COOH-MIC and NH_2_-MIC (data not shown). The ζ-potential measurements performed on MIC, MIC-COOH and MIC-NH_2_ samples point out an overall negative charge at the surface, reasonably ascribable to the phosphate moiety in the phospholipid structure (Fig 4B). The presence of a certain amount of amine groups at the surface of MIC-NH_2_ results in an overall charge that, while remaining still negative, is more positive than that measured on MIC and MIC-COOH. Such an evidence is reasonable and consistent considering that the PEG-layer at micelle surface, in fact, reduces the effective number of end groups (either amine or carboxylic) due to the entanglement of the chains, which shields charges, thus resulting in an overall lower detected charge. These results demonstrated that the investigated micellar system is characterized by a net negative charge, that can be considered a beneficial aspect for the designed system. Indeed Lockman et al. [37] clearly pointed out that surface charge must be considered for toxicity and distribution profiles into the brain, since the extent of brain uptake of anionic nanoparticles is higher to that observed for neutral or cationic formulations, at the same concentrations [38]. Cytotoxicity studies were performed by incubating astrocytes with MIC, MIC-COOH and MIC-NH_2_ solutions at lipid concentrations ranging from 0.5 to 25 μM for 24 hours (Fig 5). The microscopical observation of astrocytes does not show any significant difference between the untreated control and the cells treated with the three different preparations of PEG-lipid micelles (Fig 5). In particular for concentration values below 12.5 μM no difference was detected between cells treated with the three different PEG-lipid micelle preparation and control cells. By contrast, for the 12.5 μM micelle preparations, the astrocytes treated with the differently terminated empty PEG-lipid micelles were reduced in number while the exposure to empty PEG-lipid micelle at concentration of 25 μM induced cell death (Fig 5). The comprehensive studies on PEG-PE based micelle interaction with cells, reported by J. Wang et al. [39,40] demonstrated that PEG-PE micelles can insert into cell membrane without affecting their integrity, as micelles disassembly, and the spare PEG-PE molecules selectively accumulate in Golgi apparatus and endoplasmatic reticulum (ER), inducing ER stress. In addition, anionic nanoparticles are known to induce intracellular and not just membrane damage [41].

Therefore, here it is reasonable to assume that the here observed toxicity of the negatively charged micelles, mainly or only formed of PEG-PE, with a lipid concentration above 12.5 μM, comes from the disturbance of ER membrane lipid homeostasis due to an accumulation of PEG-PE in the ER, upon their cellular internalization which may also occur without disrupting the membrane integrity [39,40]. Furthermore, the data on cell viability, obtained upon exposure to the three different preparations of PEG-lipid micelles and assessed by the MTT assay, revealed that the MIC-NH_2_ were toxic at concentration above 2.5 μM (IC50 = 4.183 μM; 95% confidence interval = 3.29 to 5.072 μM), whereas both MIC and MIC-COOH displayed cell toxicity at concentrations above 5 μM (IC50 = 6 μM, 95% confidence interval = 5.9–7.65 μM for MIC and IC50 = 7.9 μM, 95% confidence interval = 6.23–9.55 μM for MIC-COOH) (Fig 5B). The correlation analysis between the surface charge obtained by ζ-Potential measurements (Table B, Fig 4) and the cell viability (Fig 5B) shows that the toxicity of empty PEG-lipid micelles is dependent on the surface charge induced by the terminal functional groups (r^2^ = 0.99). In particular, the cell viability decreases with a reduction of the negative charges on the surface of PEG-lipid micelles. Therefore, the toxicity of only the NC containing COOH terminated PEG-modified lipid micelles (NC/MIC-COOH), characterized by a higher negative surface charge, was subsequently investigated.

### Assessment of nanocrystal containing PEG-lipid micelle toxicity on cell viability of astrocytes

**Fig 6:**
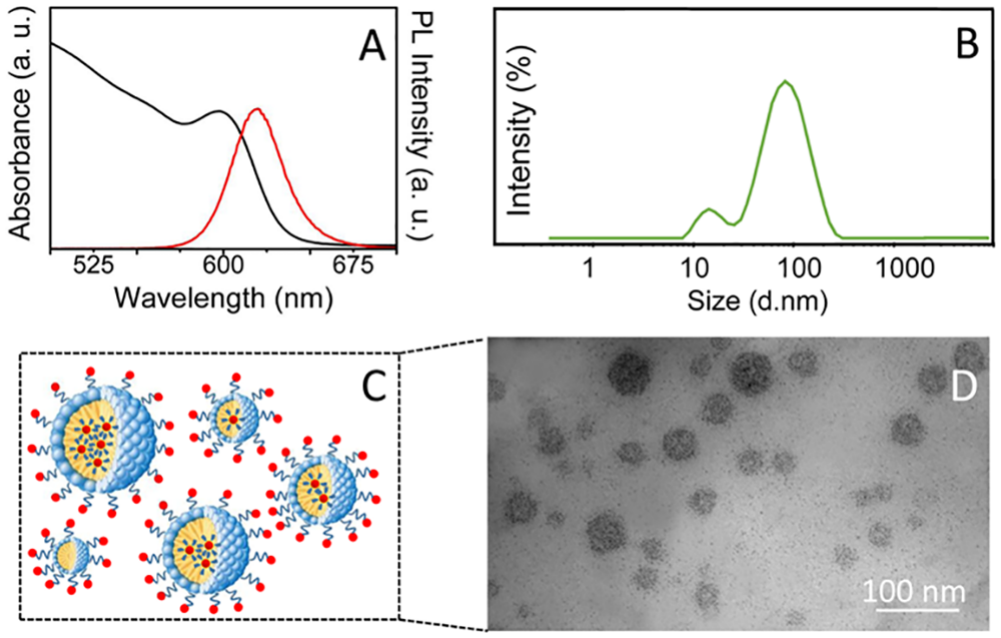
Preparation and characterization of NC/MIC-COOH. Absorption (black line), PL (red line) spectra (A), hydrodynamic diameter distribution by intensity (B) and TEM micrograph with positive staining (D) of NC/MIC-COOH dispersed in PBS at pH 7.4. Schematic sketch of NC/MIC-COOH (C).

Toxicity effect of NC containing COOH terminated PEG-lipid micelles (NC/MIC-COOH) on astrocytes was assessed. The NC encapsulation in the core of the micelles was achieved by exploiting hydrophobic interactions between the pristine capping ligand of luminescent NCs, namely TOPO and TOP and the hydrophobic tails of lipids [21,22]. Interestingly, the optical characterization of NC/MIC-COOH performed by means UV-Vis and PL spectroscopy revealed that the encapsulation process in PEG lipid micelles allows to retain the CdSe@ZnS core-shell NC spectroscopic properties (Fig 6A), and, in particular, the size dependent NC visible emission, fundamental for their use in optical bioimaging. DLS investigation resulted in a bimodal size distribution for NC/MIC-COOH (Fig 6B), indicating that small aggregates, with size compatible with that of the empty micelle, concomitantly form during the micellization process (Fig 4C), and coexist with larger aggregates (Fig 6B). In particular, average hydrodynamic diameters of 20.1 ± 3.7 and 85.7 ± 9.2, respectively, were recorded for the small and large aggregates, respectively (PDI = 0.189±0.030).

The population with larger average hydrodynamic diameter can be ascribed to clusters of a certain number of NCs embedded within one micelle, as also previously reported in literature [22]. This result was confirmed by TEM investigation which clearly indicates that the micelle diameter ranges from 15 to 80 nm, pointing out the formation of aggregates in a wide range of sizes, containing a variable number of NCs clustered in a single micelle (Fig 6C and 6D). The occurrence of such micelles containing several luminescent NCs each can be indeed seen as beneficial for a detection in biological systems, being more sensitive than a single NC micelle system, as they demonstrated an increased sensitivity for *in vitro* and *in vivo* imaging [42]. The ζ-potential measurements performed on NC/MIC-COOH resulted in a value of 16.7 ± 0.7 mV, thus confirming an overall negative charge at their surface and indicating a good colloidal stability.

**Fig 7:**
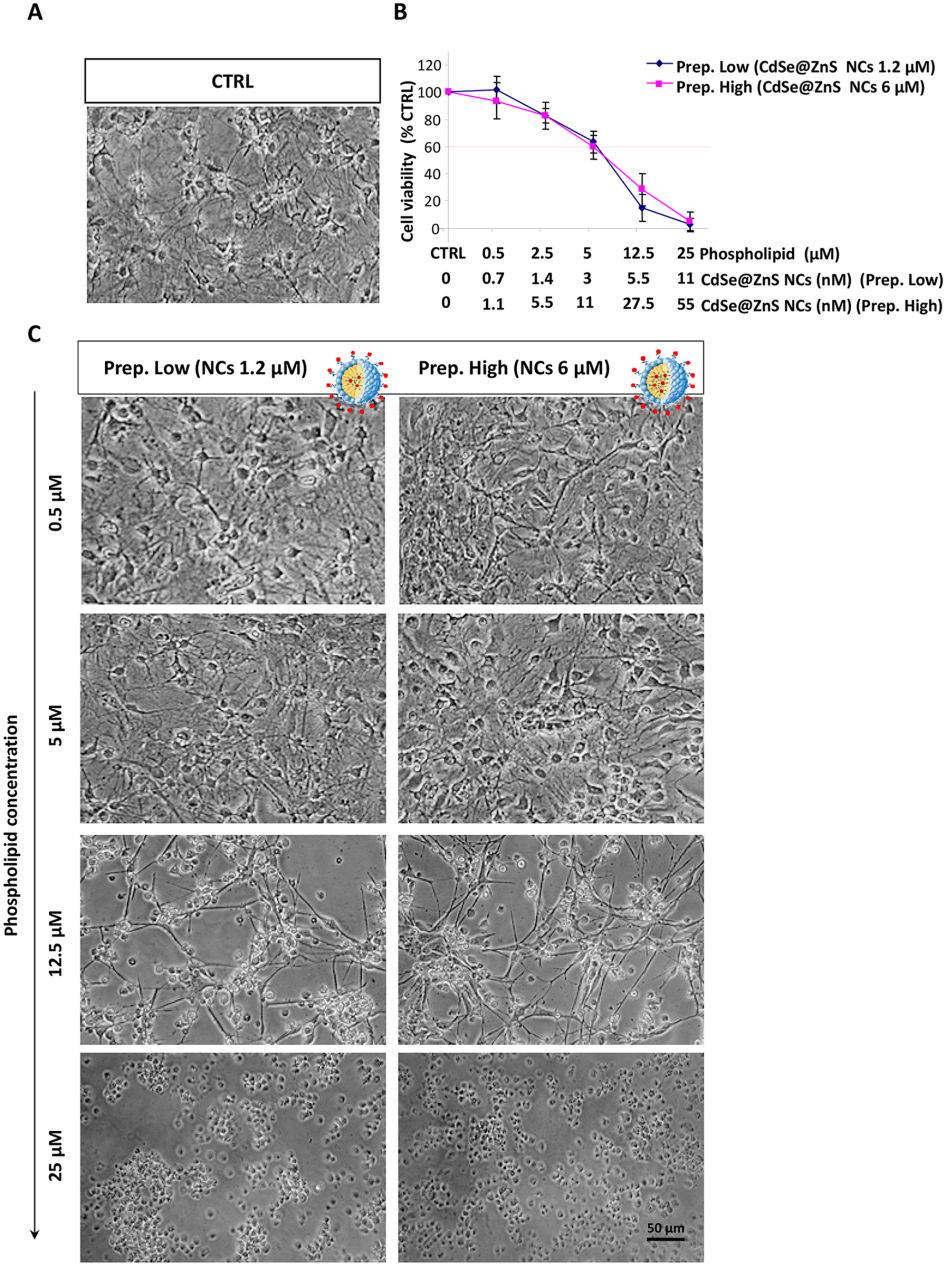
Effect of CdSe@ZnS NC containing PEG-lipid micelles on cell viability of astrocytes. Confluent astrocytes, plated in 96 well plates, were treated, at the indicated phospholipid concentrations, with two different preparation of PEG-lipid micelle functionalized with COOH-groups and containing CdSe@ZnS NCs at concentration of 1.2 μM (Prep. Low) and 6 μM (Prep. High) respectively (NC/MIC-COOH). The control (CTRL) was obtained from untreated astrocytes in serum-free DMEM. After treatment for 24 h at 37°C, 5% CO_2_ the cells were subjected to the cell viability test with MTT as described in Experimental section. Micrographs show representative results of cell morphology observed under phase-contrast microscope (50X magnification) after 24 h of treatment (A and C). The graphs represents the cell viability expressed as percentage of survival cells in comparison with control (CTRL) (B). The doses of NC/MIC-COOH that determined a cell viability < 60% were considered toxic. Data represent the mean values ± SD of three experiments performed on different cell populations.

The effect of NC/MIC-COOH on cell viability was tested by treating astrocytes with two different preparations of luminescent NC/COOH-MIC, with a final fixed phospholipid concentration ranging between 0.5–25 μM. The two preparations of NC/COOH-MIC differing for the NC content, were used to incubate the cells, namely the low NC concentration sample allowed to explore a final concentration range between 0–11 nM, while the high NC concentration preparation the range between 0–55 nM. No significant morphological difference in the cells treated with the two preparations of NC/COOH-MIC, respectively, were detected by microscopic observation. In both cases a morphology comparable to that observed for astrocytes treated with empty PEG-lipid micelles was observed. In particular, the cells treated with the two preparations of NC/COOH-MIC at phospholipid concentrations up to 5 μM, showed a morphology similar to that of control, while at phospholipid concentration of 12.5 μM fewer and more ramified astrocytes were observed. Cells treated with NC/COOH-MIC at lipid concentration of 25 μM appeared with a rounded cell body without a well-defined structure (Fig 7A and 7C). This result was confirmed by the test of cell viability (Fig 7B). Indeed, astrocytes treated with the two different preparations of NC/COOH-MIC did not show significant differences in cell survival. Both preparations of NC/COOH-MIC were toxic for astrocytes at phospholipid concentration higher than 5 μM (Prep high: IC50 = 8.543 μM, 95% confidence interval = 6.63–10.45 μM; Prep low: IC50 = 8.495 μM, 95% confidence interval = 6.77–10.22 μM), which corresponded to the NC concentration of 3 nM and 11 nM, respectively, which are concentration values that conversely resulted to not affect the cell viability.

In this regard, the above reported cytotoxicity studies performed on the *‘as synthesized’* luminescent NCs demonstrated that NCs at concentrations below 25 nM did not induce toxicity for astrocytes (Fig 3). Therefore, it is reasonable to assume that, under the investigated conditions, cell viability of astrocytes is mainly affected by the final concentration of phospholipids, rather than that of the NCs embedded in the PEG-modified lipid micelles. This assumption is corroborated by the two different cell viability data set obtained incubating the cells with the two preparations of NC/COOH-MIC, at the same NC concentration (11 nM) but at different lipid concentrations, thus highlighting that the toxicity to astrocytes essentially depends on the lipid concentration. This result is encouraging because it suggests that, under the investigated experimental conditions, the CdSe@ZnS NCs incorporated in micelles may be used without inducing toxic effects. Therefore, even assuming that NC/COOH-MIC are internalized by cells and that the micelles can break releasing NCs, such as reported in literature [36,37], these should not be harmful for astrocytes at the actual NC concentration.

### Evaluation of cellular uptake of nanocrystal containing PEG-lipid micelles by epi-fluorescence and confocal microscopy

**Fig 8:**
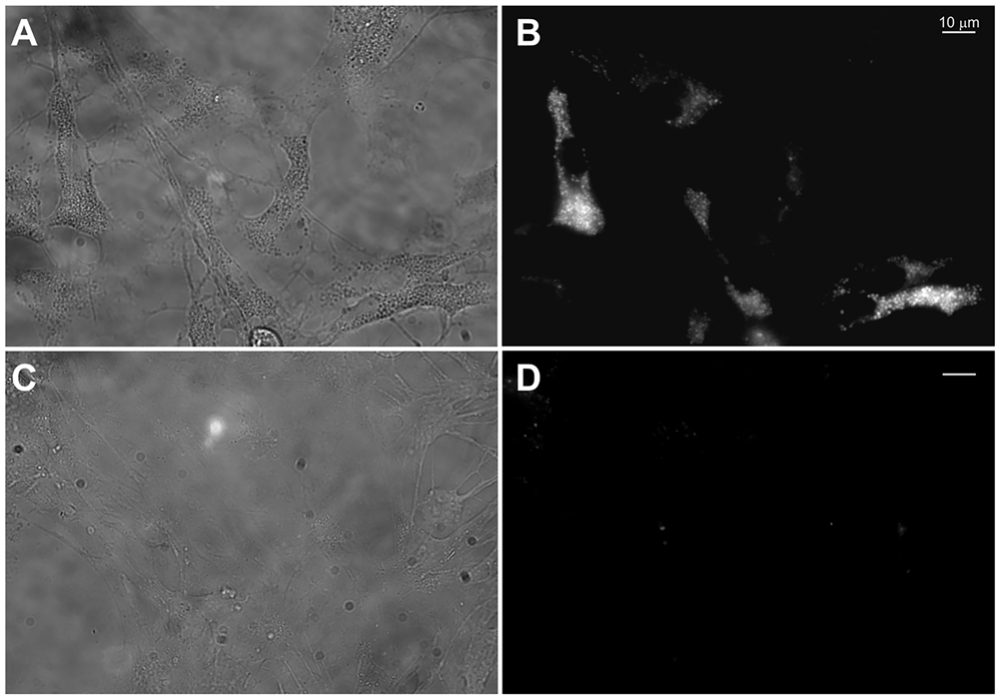
Intracellular visualization of NC/MIC-COOH nanoparticles by live cell epi-fluorescence microscopy. Representative bright field (A, C) and fluorescence (B, D) images of living astrocytes after 9 h of incubation with NC/MIC-COOH at the NC concentration of 0.2 nM (A, B) or with serum-free medium alone (negative control) (C, D).

The subcellular localization of NC containing PEG-Lipid micelles (NC/MIC-COOH) was qualitatively investigated in primary rat astrocytes by epi-fluorescence and confocal microscopy. Astrocytes, cultured on glass coverslips coated with poly-L-lysine (PLL), were treated for 9 hours with a preparation of NC/MIC-COOH at the final phospholipid concentration of 200 nM and at a corresponding NC concentration of 0.2 nM. Live cell imaging measurements clearly demonstrated that, even at this very low NC concentration, luminescent NCs were clearly detectable in intracellular vesicles localized throughout the cytoplasm and excluded from the nuclear region (Fig 8, panel B) as previously described by others in different cell types [43]. As evident from the bright field image in panel A of Fig 8, astrocytes incubated in these experimental conditions did not show any morphological evidence of cell suffering and resulted fully viable.

**Fig 9:**
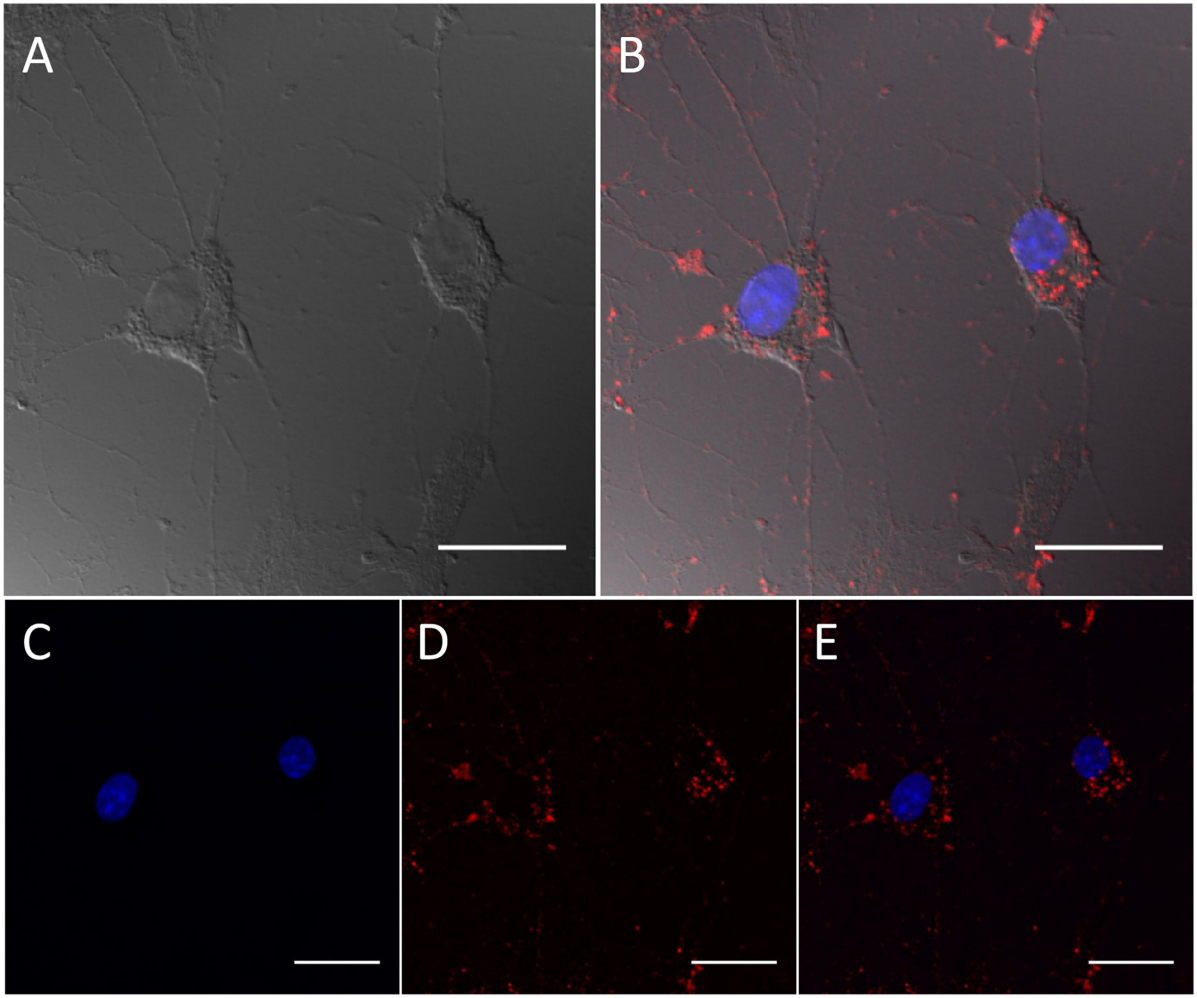
Evaluation of cellular uptake of NC/MIC-COOH by confocal microscopy. Confocal differential interface contrast and fluorescence micrographs of fixed astrocytes. Cells images after 1 h of incubation time with NC/MIC-COOH at NC concentration of 0.2 nM. Cell images in the differential interference contrast (Panel A), blue (Panel C) and red (Panel D) detection channel. Overlay of blue and red fluorescence detection channels with (Panel B) and without differential interface contrast (Panel E). Scale bar 25 μm.

Further evidence of the effective uptake of NC/MIC-COOH in astrocytes was demonstrated by confocal microscopy investigation (Fig 9). Cells were incubated with NC/MIC-COOH at the final phospholipid concentration of 2 μM (corresponding to a NC concentration of 0.2 nM) for 1 hour, fixed and treated with Hoechst 33258 to stain cell nuclei. The blue and red PL of Hoechst and luminescent NCs are shown in panel B and C of Fig 9, respectively. The occurrence of blue and red emission is evident in the overlay of panel D. Such fluorescence microscopy experiments clearly confirm the ability of NC/MIC-COOH to enter primary rat astrocytes and their detectability even at very low NC concentrations. Conversely, confocal microscopy investigation performed on cells incubated with *‘as synthesized’* NCs, under the same experimental conditions tested for NC/MIC-COOH (NC concentration of 0.2 nM and time incubation of 1 hour), clearly indicates lack of any PL signal in the red channel, where emission due to the NC presence in the cells would have been, in fact, detected (S1 Fig). This observation proved that the *‘as synthesized’* red emitting NCs are not able to be internalised by the cells. On the contrary, it can be reasonably supposed that, according to previous reports by Jiang Wang et al. [39,40], the uptake of NC/MIC-COOH likely occurs via non-specific extensive endocytosis, similarly to what observed for empty or drug-loaded PEG-PE micelles. Thus, it can be assumed that, in agreement with other experimental models, after insertion into astrocytic plasma membrane, spare PEG-phospholipids and NCs are released intracellularly, and therein the luminescent NCs accumulate as red emitting vesicles, however not reaching the nucleus (Figs 8 and 9).

### Detection of nanocrystal containing PEG-lipid micelle fluorescence in cell lysates

**Fig 10:**
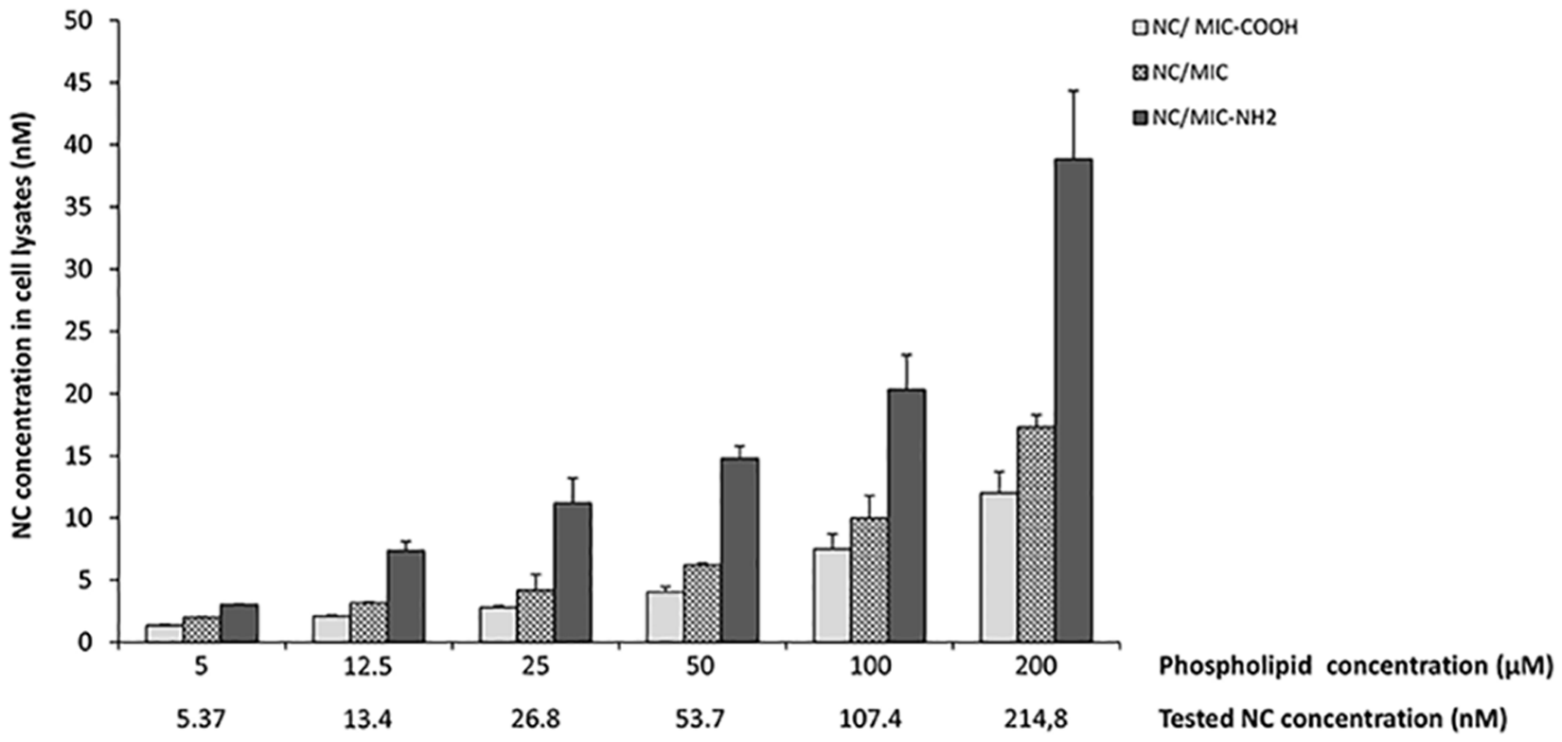
Concentration of emitting CdSe@ZnS NCs in cell lysates. Confluent astrocytes plate in 6 well plates, were treated for 1h with NC/MIC, NC/MIC-COOH or NC/MIC-NH_2_, at the reported concentrations of phospholipids and NCs. Negative control, obtained from untreated astrocytes in serum free DMEM, was set at zero. After incubation, the concentration of fluorescence CdSe@ZnS NCs in cell lysates was determined by spectrophotometric assay as reported in experimental section. The histograms represent the NC concentration in cell lysates, expressed as mean values ±SD of three experiments performed on different cell populations.

The quantitative evaluation of the cellular uptake of the three different PEG-modified phospholipid micelles loaded with NCs, namely NC/MIC, NC/MIC-NH_2_ or NC/ MIC-COOH in astrocytes was carried out by recording NC emission in cell lysates. For this purpose, astrocytes were treated with NC/MIC, NC/MIC-NH_2_ or NC/MIC-COOH, for 1 hour at the phospholipid concentration of 5, 12.5, 25, 50, 100 and 200 μM. During the incubation with luminescent NCs, cells were monitored under a phase-contrast microscope to assess their viability and, after the appropriate incubation time, cells were lysed. Subsequently, an amount of cell lysate corresponding to 500 μg of proteins was subjected to spectrofluorimetric assay, as described in Experimental section. Fig 10 report the concentrations of NCs in lysates from astrocytes treated with the tree different types of NC containing micelles at the reported final concentrations of NCs and phospholipids.

As evidenced in Fig 10, the three different types of NC containing micelles were internalized into the cells after 1hour of incubation and, interestingly, astrocytes showed no morphological sign of cell suffering also at the highest tested concentrations (data not shown).

It is worth to note that, also in this set of experiments, at very low NC exposure concentration, the emitting NCs, incorporated into the micelles, are still detectable and dosable within the cell lysates. In addition, the results of these experiments clearly demonstrate that the uptake of micelles is dependent from the surface charge of the terminal functional groups. In fact, in the same experimental conditions the micelles with NH_2_ functional groups exhibited the highest uptake. In particular, the uptake increased with the reduction of negative charges on the surface of PEG-lipid micelles. Indeed, the presence of amine groups at the surface of NC/MIC-NH_2_ results in a net charge (-16.7 ± 0.7 mV) that, while remaining still negative, is more positive than that measured on surface of NC/MIC (-15.5 ± 0.5 mV) and NC/MIC-COOH (-11.2 ± 0.4 mV), such as observed for the corresponding empty micelles.

### ROS production in astrocytes treated with empty or nanocrystal containing PEG-lipid micelles

**Fig 11:**
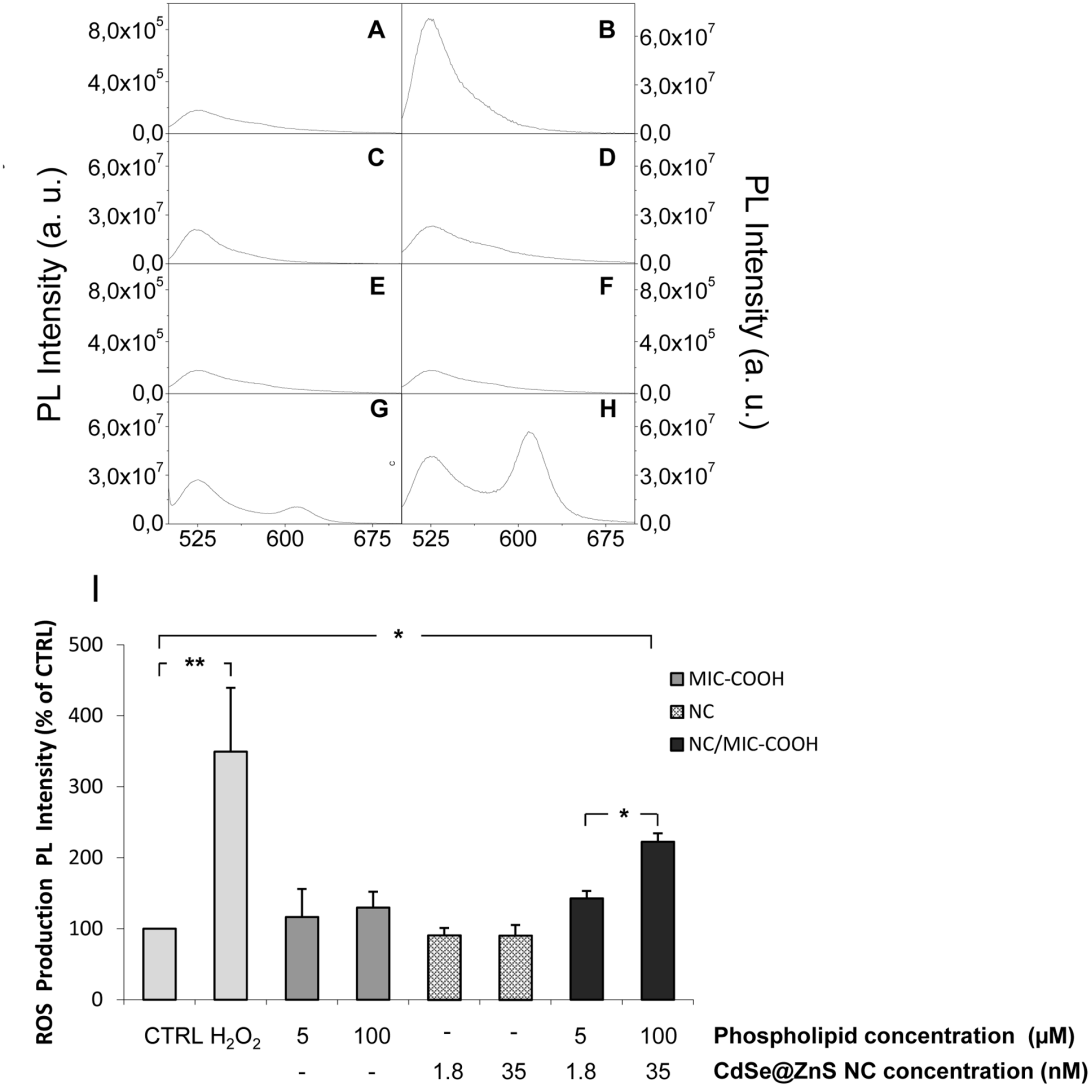
Effect of NCs, MIC-COOH and NC/MIC-COOH on the production of ROS in astrocytes. PL spectrum of the lysates from astrocytes treated only with DCFH-DA (CTRL)(A); pretreated with DCFH-DA and then treated with: 100 μM of H_2_O_2_ ((B); MIC-COOH at lipid concentration of 5 μM (C) and 100μM (D); *‘as synthesized’* NCs at concentration of 1.8 nM (E) and 35 nM (F); NC/MIC-COOH at lipid concentration of 5 μM and NC concentration of 1.8 nM (G) or NC/MIC-COOH at lipid concentration of 100 μM and NC concentration of 35 nM (H). The PL peak centred at 525 nm is ascribable to DCF, while the peak centred at 605 nm is due to luminescent CdSe@ZnS NCs. Histograms represent ROS production, reported as relative percentage of PL intensity in comparison with the negative control (I). Data are mean values ± SD of three separate experiments performed on different cell populations (one-way ANOVA followed by Student-Newman-Keuls; *p < 0.05 and **p < 0.001).

While toxicity on nanomaterials was widely assessed, still an open challenge remains to fully understand the origin and chemical interpretation of the observed toxicity, due to the complexity of both nano- and biosystems. In this perspective, among the different phenomena, generation of reactive oxygen species (ROS) by cultured cells upon exposure to nanoparticles was typically reported [44]. Sensitivity of cells to ROS may depend on cell type and levels and duration of exposure to nanoparticles. Therefore in this study ROS production was evaluated to investigate the possible involvement of an oxidative stress in NC cytotoxicity of astrocytes. In particular, the intracellular ROS level was assayed by measuring variation in the fluorescent signal intensity after the addition of a not fluorescent probe, 2′,7′-dichlorofluorescein diacetate (DCFH-DA), in astrocytes treated with *‘as synthesized’* NCs at the final concentration of 1.8 and 35 nM, or with MIC-COOH and NC/MIC-COOH at final phospholipid concentration of 5 and 100 μM, respectively. The two final NC concentrations tested for the NC/MIC-COOH samples were 1.8 and 35 nM. Cells treated only with DCFH-DA and H_2_O_2_ were used as negative (CTRL) and the positive control, respectively. As showed in the representative DCF emission spectra reported in Fig 11, low levels of ROS were detected in CTRL cells (A) and in the cells treated with MIC-COOH (C, D). Levels of ROS comparable to those of the CTRL were also detected in cells treated with both concentrations of *‘as synthesized’* NCs (E, F). Conversely, an increase in DCF-fluorescence intensity was observed upon treatment of the cells with NC/MIC-COOH containing the highest tested concentration of NCs (H). As shown in the spectra reported in Fig 11 in the lysate from astrocytes treated with *‘as synthesized’* NCs (E, F) only the peak at 525 nm, ascribable to DCF, is present while the peak at 605 nM, ascribable to NCs, is not detected. Conversely, the peak at 605 nm is visible in the lysate from astrocytes treated with NCs incorporated into the micelles (G, H) indicating that the free NCs are not able to enter the cells. The statistical analysis (I) indicated that the treatment of astrocytes with empty MIC-COOH did not induce a significant production of ROS both at the not toxic concentration of 5 μM, as well as, at the toxic concentration of 100 μM. Similarly, NC/MIC-COOH at the final phospholipid concentration of 5 μM and NC concentration of 1.8 nM, that are conditions resulted not toxic, did not induce a significant production of ROS. Conversely, a statistically significant increase in ROS production was observed in astrocytes treated with NC/MIC-COOH at phospholipid concentration of 100 μM and NC concentration of 35 nM. These evidences point out that phospholipids cannot be considered responsible of cytotoxicity induced by ROS production, thus confirming the results of Jiang Wang et al. [40], that demonstrated that PEG-PE micelles accumulate in the ER and their cytotoxicity in cancer as well as in normal cells can be ascribed to ER stress, but not to oxidative stress. In fact, we found that the production of ROS in NC/MIC-COOH treated cells is not dependent on the phospholipid concentration, but only on NC concentration.

The micelles can be assumed to break once entered the cell membrane and release the originally contained NCs in intracellular environment, resulting toxic when their concentration is high and thus able to lead to cell damage and death.

Different mechanisms were proposed to explain the cell damage. Among the others, Cd^2+^ ions, released by NCs, were reported to generate free radicals or, alternatively, NCs themselves were found to form ROS. Some experimental evidences indicated that NCs are redox active nanoparticles that can induce ROS generation through energy or electron transfer to molecular oxygen [44]. ROS can cause damage to DNA, lipids and other cellular components such as membrane of mitochondrial as a consequence induction of apoptosis and necrosis.

## Conclusions

A comprehensive and systematic investigation on the in vitro response of primary cultures of rat astrocytes exposed to luminescent CdSe@ZnS NCs provided relevant insight on their toxicity before and after their incorporation in PEG-micelles. The results provide clear evidence that *‘as synthesized’* NCs did not enter the cells and pointed out that cytotoxicity of NCs becomes significant only for concentration higher than 10 nM, probably due a higher extent of Cd^+2^ ions released at such high concentration. The incorporation of the organic capped CdSe@ZnS NCs in the hydrophobic core of pegylated phospholipid micelles demonstrated to make them stable and dispersible in aqueous media, minimizing possible Cd^2+^ release and allowing the nanostructure penetration in the cell membrane of astrocytes. Preliminarily, the investigation of toxicity of empty PEG-lipid micelles was performed and revealed that a lipid concentration of 25 μM induced cell death. In addition the toxicity of empty PEG lipid micelles, tentatively ascribed to ER stress, was found dependent on the nature of the PEG micelle end group, and the micelles with carboxylic groups turned out as less toxic. Interestingly, test performed on two different preparations of NPs, based on micelles functionalized with carboxylic groups, at a low and a high-close to the value found toxic to the astrocytes-NC concentration, respectively, showed the same toxicity behavior towards astrocytes, thus highlighting that the concentration of lipid, rather than that of the incorporated NCs, is a critical issue in terms of toxicity towards cell culture of astrocytes. Finally, the NCs incorporated into the micelles, although at a very low concentration, below the value resulted cytotoxic for astrocytes, demonstrated still detectable within the cells.

Finally, the generation of ROS induced by NCs incorporated into micelles functionalized with carboxylic groups was found not dependent on the phospholipid concentration, but only on NC concentration, thus confirming that cytotoxicity of PEG-modified phospholipids can be ascribed to ER stress and not to oxidative stress, while cytotoxicity of NCs is mainly due to ROS production.

The overall investigation thus provided relevant information on the experimental conditions for using of luminescent NC incorporating PEG-modified lipid micelles as relevant nanostructures with very good stability and dispersibility in biological media, for *in vivo* labelling and diagnostic studies.

## Supporting Information

S1 Fig Confocal differential interface contrast and fluorescence micrographs of fixed astrocytes. This figure provides a clear evidence of the ineffective uptake of *‘as synthesized’* CdSe@ZnS NCs in astrocytes by confocal microscopy investigation. Cells were incubated with *‘as synthesized’* CdSe@ZnS NCs at the final NC concentration of 0.2 nM for 1 hour, fixed and treated with Hoechst 33258 to stain cell nuclei. Cell images in the differential interference contrast, red, blue detection channel are reported in the panel A, B and D respectively. Overlay of blue and red fluorescence detection channels with differential interface contrast is shown in panel D. Confocal microscopy images reported in panel B clearly indicate lack of any PL signal in the red channel, where emission due to the NC presence in the cells would have been, in fact, detected. This observation proved that the *‘as synthesized’* red emitting NCs are not able to be internalised by the cells.
(TIF)
