# Supplementary figures and images for: Cytotoxicity Study on Luminescent Nanocrystals Containing Phospholipid Micelles in Primary Cultures of Rat Astrocytes
Source: PLoS One. 2016 Apr 20;11(4):e0153451. doi: 10.1371/journal.pone.0153451 (PMC4838222; doi:10.1371/journal.pone.0153451)

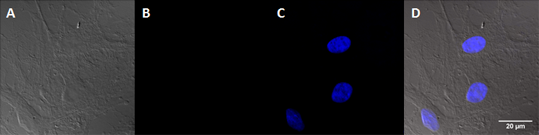

Supplement: S1 Fig — This figure provides a clear evidence of the ineffective uptake of ‘as synthesized’ CdSe@ZnS NCs in astrocytes by confocal microscopy investigation. Cells were incubated with ‘as synthesized’ CdSe@ZnS NCs at the final NC concentration of 0.2 nM for 1 hour, fixed and treated with Hoechst 33258 to stain cell nuclei. Cell images in the differential interference contrast, red, blue detection channel are reported in the panel A, B and D respectively. Overlay of blue and red fluorescence detection channels with differential interface contrast is shown in panel D. Confocal microscopy images reported in panel B clearly indicate lack of any PL signal in the red channel, where emission due to the NC presence in the cells would have been, in fact, detected. This observation proved that the ‘as synthesized’ red emitting NCs are not able to be internalised by the cells. (TIF) [file pone.0153451.s001.tif]
